# Individualized targeted treatment in a case of a rare *TFG::ROS1* fusion positive inflammatory myofibroblastic tumor (IMT)

**DOI:** 10.1002/cnr2.1916

**Published:** 2023-11-11

**Authors:** Sebastian Sommer, Maximilian Schmutz, Tina Schaller, Patrick Mayr, Sebastian Dintner, Bruno Märkl, Ralf Huss, M. Monika Golas, Michaela Kuhlen, Frank Jordan, Rainer Claus, Bernhard Heinrich

**Affiliations:** ^1^ Department of Hematology and Oncology Faculty of Medicine, University of Augsburg Augsburg Germany; ^2^ General Pathology and Molecular Diagnostics, Faculty of Medicine University of Augsburg Augsburg Germany; ^3^ Human Genetics, Faculty of Medicine University of Augsburg Augsburg Germany; ^4^ Pediatrics and Adolescent Medicine, Faculty of Medicine University of Augsburg Augsburg Germany; ^5^ Swabian Children's Cancer Center University Medical Center Augsburg Augsburg Germany; ^6^ Comprehensive Cancer Center Augsburg (CCCA), Faculty of Medicine University of Augsburg Augsburg Germany; ^7^ Heinrich/Bangerter Hämatologie‐Onkologie im Zentrum MVZ Augsburg Germany

**Keywords:** crizotinib, inflammatory myofibrolastic tumor (IMT), personalized oncology, *ROS1* fusion, targeted therapy, TKI

## Abstract

**Background:**

Inflammatory myofibroblastic tumor (IMTs) are rare mesenchymal neoplasms with slow growth. Resection is considered as therapeutic standard, with chemotherapy being insufficiently effective in advanced disease. *alk* translocations are present in 50% of cases, *ROS1* fusions (*YWHAE::ROS1, TFG::ROS1*) are extremely rare. Here, we present a case with *TFG::ROS1* fusion and highlight the significance of molecular tumor boards (MTBs) in clinical precision oncology for post‐last‐line therapy.

**Case Presentation:**

A 32‐year‐old woman presented with IMT diagnosed at age 27 for biopsy and treatment evaluation. Previous treatments included multiple resections and systemic therapy with vinblastine, cyclophosphamide, and methotrexate. A computed tomography scan showed extensive tumor infiltration of the psoas muscles and the posterior abdomen. Next generation sequencing revealed an actionable *ROS1* fusion (*TFG::ROS1*) with breakpoints at exon 4/35 including the kinase domain and activating the RAS‐pathway. *TFG*, the Trk‐fused gene, exerts functions such as intracellular trafficking and exhibits high sequence homology between species. Based on single reports about efficacy of ROS1‐targeting in *ROS1* translocation positive IMTs the patient was started on crizotinib, an ATP‐competitive small molecule c‐MET, ALK and ROS1‐inhibitor. With a follow‐up of more than 9 months, the patient continues to show a profound response with major tumor regression, improved quality of life and no evidence for severe adverse events.

**Conclusion:**

This case underscores the importance of the availability of modern molecular diagnostics and interdisciplinarity in precision oncology to identify rare, disease‐defining genotypes that make an otherwise difficult‐to‐treat disease targetable.

## 1 INTRODUCTION

Inflammatory myofibroblastic tumors (IMTs) are rare mesenchymal neoplasms, with approximately 150–200 new cases diagnosed each year in the United States.^1^ They belong to the group of inflammatory pseudotumors and represent a distinct pathological entity. While they predominantly manifest in children at an average age of 9–10 years, in adolescents, cases have been reported across all age groups. IMTs often form in the retroperitoneum, pelvis, abdomen, and lungs, but can arise in any other anatomic location.^2^

Histopathologically, IMTs are defined by a variable proliferation of spindle cells on both myxoid or collagenous stroma with prominent infiltrates of plasma cells and lymphocytes.^2^ Three major histological patterns have been defined: (1) areas with myxoid, vascular, and inflammatory features resembling nodular fasciitis; (2) infiltrates of inflammatory cells within compacted spindle cells with resemblance to fibrous histiocytoma; (3) plate‐like patterns of collagen with resemblance to a desmoid scar.^3^

Molecular characterization of IMTs demonstrated that every second case harbors a translocation of the *ALK* gene.^4^ The 3′ kinase region of ALK can be fused to several partners, among them are *TPM4*, *CLTC*, *CARS*, *ATIC*, *SEC31L1*, *PPFIBP1*, *DCTN1*, *EML4*, *PRKAR1A*, *LMNA*, *TFG*, *FN1*, *HNRNPA1*.^5^, ^6^, ^7^, ^8^, ^9^, ^10^, ^11^, ^12^, ^13^ *ALK* fusion negative cases are much rarer and include translocations of *ROS1*, *PDGFRB*, *NTRK3*, *RET*, and *IGF1R*.^7^, ^14^, ^15^

Clinically, IMTs are characterized by slow, non‐aggressive growth patterns. Constitutional symptoms are often the cause for primary presentation to a health care provider. The standard treatment approach for localized disease is complete resection and has high potential for cure. Although metastatic disease is extremely rare, 25% of patients with abdominopelvic tumors experience local recurrence.^3^ In patients with advanced or metastatic disease, therapeutic options include chemotherapy, tyrosine kinase inhibitors (TKI), and nonsteroidal anti‐inflammatory drugs (NSAIDS). Retrospective studies demonstrate a high degree of efficacy with overall response rates (ORR) ranging from 48% to 64% for classical chemotherapy. Successfully applied regimens include adriamycin and ifosfamide, methotrexate, vinblastine, gemcitabine, docetaxel, and cyclophosphamide.^16^, ^17^, ^18^ The use of NSAIDs has also been reported, including combinations with methotrexate or methotrexate/cisplatin.^19^, ^20^

For *ALK* mutated IMTs, ALK inhibitors are currently the preferred treatment option. Based on case reports, a phase I/II study with crizotinib was initiated, which showed an overall response rate (ORR) of 50% (6 of 12 patients) and a disease control rate (DCR) of 100%.^21^, ^22^ In a separate phase IB study, an ORR of 71% (5 of 7 patients) was reported.^23^ Data for targeted therapies in ALK‐negative patients remains scarce and represents a relevant clinical need.

## 2 CASE DESCRIPTION

Here, we describe the case of a 32‐year‐old woman with no previous medical history who presented with recurrent retroperitoneal IMT in May 2022. Prior exposure to radiation or toxins was not reported. The patient was originally diagnosed and treated in Ukraine. Retroperitoneal IMT was diagnosed in 2017 at the age of 27 years at the University Hospital in Kyiv. Treatment consisted of repeated resections in June and December 2017 as well as in August 2018. In 2018, metronomic chemotherapy was started with the NSAID celecoxib followed by vinblastine, cyclophosphamide and methotrexate. Best response was stable disease (SD). The therapy was continued until the patient fled from Ukraine in January 2022.

**FIGURE 1:**
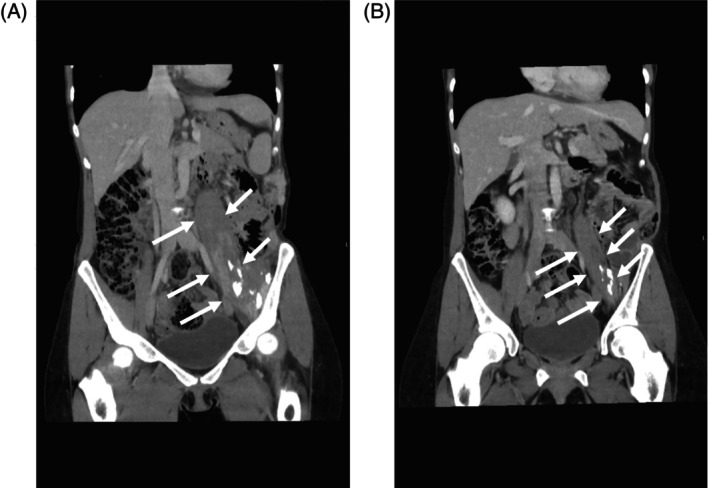
Computed tomography imaging showed inhomogeneous soft tissue formations in the psoas muscle, the iliopsoas and in the lateral paravertebral musculature (indicated by arrows) before initiation of crizotinib (A) and 5 months into therapy (B). The abdominal wall metastasis on the left paraumbilical side regressed considerably regarding soft tissue involvement. Grossly flaked calcifications and a decrease in volume, but no pathologic uptake of contrast remained in the affected muscular structures.

The patient presented to a cooperating oncology practice of the University Hospital Augsburg (UKA) with pain and functional impairment in the left gluteal region and edematous swelling of the left leg. Computed tomography (CT) scans of the abdomen showed an extensive tumorous mass infiltrating the psoas and iliopsoas muscles and subsequently infiltrating the posterior abdominal wall, lateral paravertebral muscles, and kidney (Figure 1A).

**FIGURE 2:**
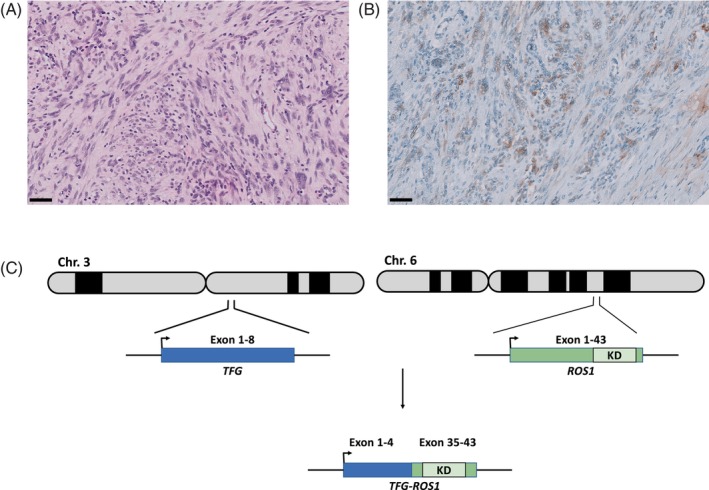
(A) Infiltration of muscle fibers by a spindle‐like mesenchymal tumor (hematoxylin and eosin stain, ×20), (B) ROS1‐antibody staining shows subtle but distinct positivity for ROS1 (×20). Scale bar = 50 μm. (C) Schematic representation of the *TFG*::*ROS1* translocation. The *ROS1* gene is located on chromosome 6 (6q22.1). *TFG* is located on chromosome 3 (3q12.2). Exons 36–41 encode the kinase domain. The four major intronic breakpoints are within the introns 31, 33, 34, and 35. *TFG* exon 1–4 are fused in‐frame to *ROS1*.

Because no tissue samples were available for additional molecular testing, a CT‐guided biopsy was performed, and the case was referred to the Molecular Tumor Board (MTB) at UKA. Histologic examination confirmed the presence of IMT (Figure 2A,B). Using the AmpliSeq for Illumina Focus Panel, RNA Sequencing of the FFPE‐Material identified a *TFG*::*ROS1* translocation (Figure 2C).

As next‐line treatment, the kinase inhibitor crizotinib was recommended, consistent with an evidence level of m1C according to National Center for Tumor Diseases (NCT) guidelines.^24^ The patient began treatment with crizotinib but rapidly developed relevant side effects. Due to edema, nausea, dizziness, and muscle cramps, the maximum tolerated dose was reduced to 250 mg daily, as opposed to the recommended dose of 250 mg twice daily. Concomitant medication consisted of dexamethasone, ondansetron, metoclopramide and metamizole.

Follow‐up with CT 5 months after therapy onset showed an excellent response with significant regression of soft tissue formations in all affected muscles (Figure 1B). The abdominal wall metastasis also markedly regressed in terms of soft tissue involvement. Flake‐like calcifications and visible volume decrease remained, but no pathological contrast uptake was detected. The imaging‐based response was associated with a significant reduction in pain, decrease in swelling of the left leg, and functional improvement of the patient. The patient is still undergoing treatment 9 months after initiation.

### 2.1 *TFG::ROS1*‐fusion

*ROS1* encodes a receptor tyrosine kinase whose physiological role in homo sapiens ultimately remains unelucidated.^25^ The neural epidermal growth factor‐like like 2 (NELL2) protein, has recently been shown to bind to the extracellular domain of mouse ROS1.^25^ NELL2 is a lumicrine factor that is secreted by testicular germ cells and mediates lumicrine signaling, an essential pathway for male fertility.^25^ Activated ROS1 signaling autophosphoryla*tes* tyrosine residues in the intracellular domain. Phosphorylated sites are consecutively accessed by SH2 domain‐containing or further canonical adaptors. These adaptor proteins then stimulate signaling via RAS‐RAF‐MEK‐ERK, PI3K‐AKT‐mTOR and JAK‐STAT3 pathways, which are mainly involved in the regulation of cell survival, growth and proliferation.^26^

Up to 55 different 5′‐gene partners have been described as fused to the 3′ region of *ROS1*. This marked heterogeneity of *ROS1* partner genes has been observed between patients and especially between different cancer types. In IMTs, *YWHAE::ROS1* and *TFG::ROS1* are the predominant translocations and make up 10% of all IMTs.^7^, ^27^, ^28^

Both translocations result in a loss of the large extracellular domain as well as a fusion (in‐frame) of the N‐terminal portion of the fusion partner with the intracellular kinase domain. Tropomyosin‐receptor kinase fused gene (*TFG)* is a common fusion partner of *ROS1*, *MET*, *ALK*, *NRTK1*, and *NTRK3*.^29^ *ROS1‐*fusions lead to constitutive activation of the kinase domain, which drives oncogenesis via the described signal transduction pathways.

**FIGURE 3:**
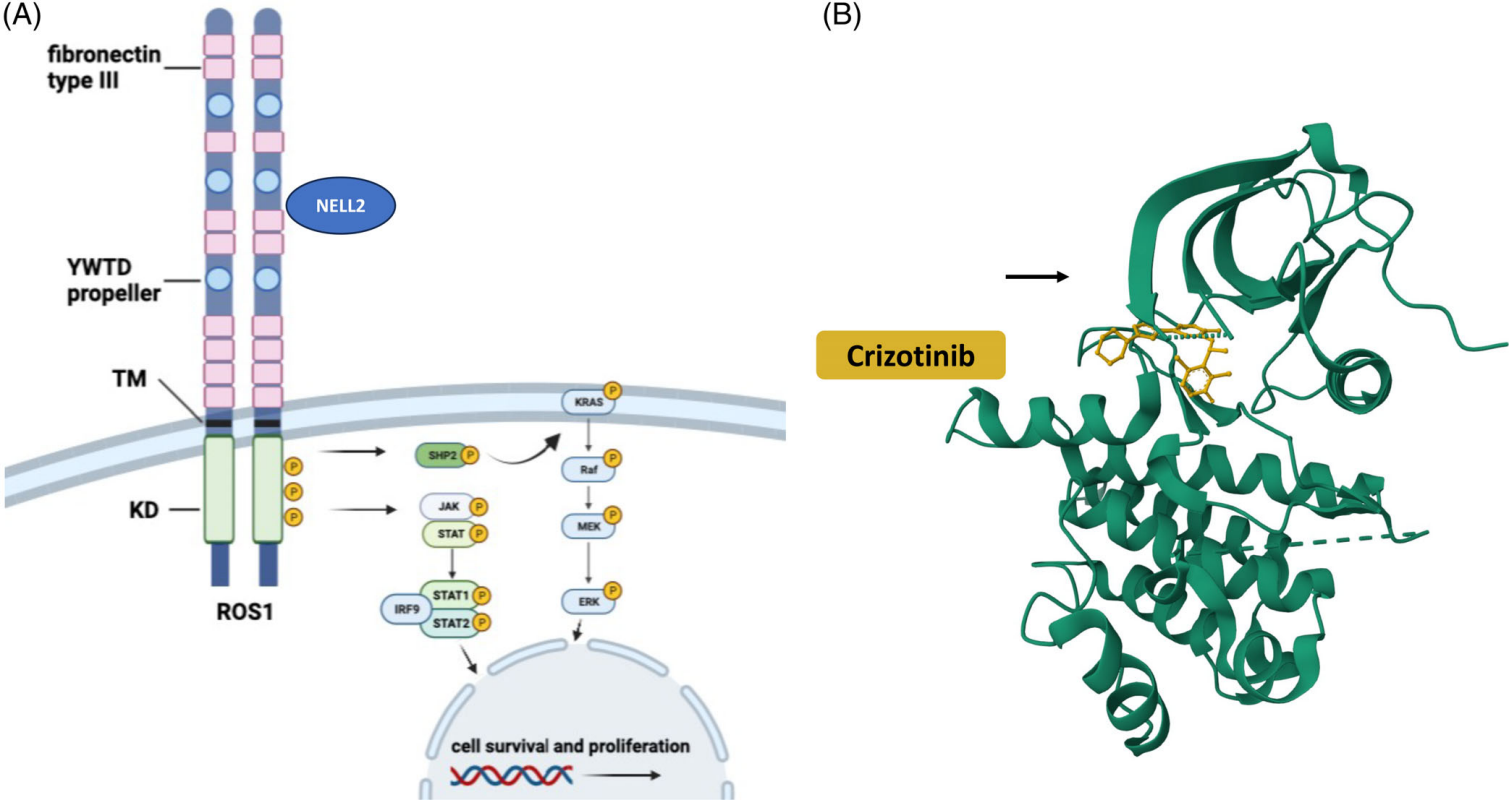
(A) ROS1 receptor domain comprises an intracellular kinase domain (KD), a transmembrane domain (TM), nine fibronectin motifs and three ß‐propeller domains. Interaction with NELL2 is supposed to mediate autophosphorylation and KD activation. Signal transduction is ultimately mediated via JAK/STAT and RAS–RAF–MEK–ERK pathways, respectively. (B) DFG‐motif in type I conformation (DFG‐in) with crizotinib shown in yellow (adapted from PDB 3ZBF).

The kinase domain of *ROS1* shares 70% homology with the ALK kinase domain. The ROS1 kinase exhibits two main conformations: type I is the active form, while type II is the inactive form. Most TKIs (e.g., crizotinib, entrectinib, ceritinib, ensartinib, brigatinib, lorlatinib, repotrectinib, and taletrectinib) bind to the active type I kinase form, which contains the ATP‐binding pocket of the ROS1 kinase, and thus inhibit its kinase activity.^26^ The type I conformation provides access to the catalytic site of the kinase domain by geometrically enabling contact through folding/orientation of an aspartic acid‐phenylalanine‐glycine motif (DFG‐motif or DFG‐in). In type I configuration, phosphatases of ATP are optimally positioned to facilitate phosphate transfer and therefore exerting catalytic activity. Type I inhibitors compete with ATP binding. In contrast, the inactive, type II, “DFG‐out” kinase domain conformation is characterized by blocked access to the catalytic site. Type II ROS1 inhibitors such as cabozantinib and foretinib preferentially bind to the type II conformation. Figure 3A,B gives an overview of the mechanism of action of crizotinib and summarizes the pathways activated by *ROS1* (adapted from^26^).

## 3 METHOD AND LIMITATIONS

Detection of the *ROS1* fusion transcript was performed by next generation sequencing (NGS) and overexpression resulting from the fusion was confirmed by immunohistochemistry. From formalin‐fixed and paraffin‐embedded (FFPE) tumor tissue, an area of the tumor was microdissected under histomorphological control with a tumor purity of 70%. Libraries were prepared from RNA and DNA using the AmpliSeq for Illumina Focus Panel amplicon based chemistry.^30^ RNA sequencing was carried out with an average on‐target aligned reads of 300 000. Data analysis was performed as previously described.^31^ In brief, the Illumina BaseSpace application was used for DNA/RNA amplicon analysis and comprehensive variant identification including SNVs, CNVs, indels and gene fusions. Non‐synonymous and non‐polymorphic variants were interpreted using Illumina's Variant Interpreter and cross‐checked via the Integrated Genome Viewer to rule out sequence errors. The BaseSpace Knowledge Network was used for additional data analysis.

Limitations and challenges of amplicon‐based panels include the limited scope of regions examined, as only certain sequence segments of the genome are covered. In particular, for the detection of larger structural variants and chromosomal translocations or gene fusions, as in this case, amplicon‐based panels have their limitations. Amplicon‐based panels can only map known fusion breakpoints and are therefore generally virtually unsuitable for detecting new unknown fusion partners or fusions at unusual breakpoints not covered by amplicons. Because fusion transcripts can vary widely in expression, quantification using amplicon‐based approaches can be inaccurate due to inherent amplification bias. However, the clinical relevance of fusions is often critically related to the extent of expression.

## 4 DISCUSSION

Here, we describe a case in precision medicine where a direct link between a molecularly defined target and disease progression exists in a rare cancer entity that allows for a targeted therapy. Our case report is further evidence of effective targeted TKI‐based therapy in IMTs. In addition, our case report highlights the value of a sophisticated diagnostic infrastructure and modern pipeline for individual therapy management in rare cancers. This is of particular importance in rare tumors such as IMTs, where identification of gene fusions is critical for highly effective personalized treatment with TKIs. Integrating advanced molecular diagnostics with an MTB that allows for thorough research and interdisciplinary evidence assessment based on available data is key to providing the most informed, evidence‐based, and individualized approach to specific oncological cases.

We illustrate the relevance and effectiveness of this approach in a rare case of ROS1‐translocated IMT. After several previous treatment approaches and lines of therapy, and most recently with progressive disease, the diagnosis of IMT was confirmed by core needle biopsy and a *TFG::ROS1* translocation was detected by NGS. The patient responded very well to crizotinib within a short period of time and showed a tolerable level of adverse events after dose adjustment. We identified 8 case reports with IMTs harboring a *ROS1* translocation including one case with the translocation partner *YWHAE1* (Table 1). All but one patient was started on crizotinib (entrectinib in one case). A single patient demonstrated a complete response (CR), whereas six of the eight patients showed a partial response (PR), with another patient exhibiting stable disease (SD). Subsequently, disease progression (PD) was observed in two patients during their treatment with crizotinib. One of the latter patients did not respond to subsequent therapy with ceritinib, but eventually the patient's tumor achieved a near CR with lorlatinib, a third‐generation TKI. The second patient had a rapid clinical response to brigatinib for 9 months and was subsequently switched to lorlatinib, which led to a PR allowing for complete resection of the tumor after 1 year.

**TABLE 1:** Published case reports of patients with ROS1‐translocated inflammatory myofibroblastic tumors.

Report	Translocation	Primary lesion	Age	Sex	Drug	Duration of response	Response	Comment
Comandini et al.^32^	*YWHAE1::ROS1*	Left thigh	23	Male	Crizotinib	> 3 years ongoing	CR	
Lovly et al.^7^	*TFG::ROS1*	Left lung	6	Male	Crizotinib	> 4 months	PR	
Srikanth Ambati et al.^33^	*TFG::ROS1*	Left lung	10	Female	Entrectinib	>13 cycles	PR	Patient chose active surveillance after very good PR
Carcamo et al.^34^	*TFG::ROS1*	Chest wall	16	Male	Crizotinib	8 months	PR	
					Ceritinib	No response		
					Lorlatinib	> 11 months	PR	Near‐complete response
Ingly et al.^35^	*TFG::ROS1*	Right Lung	14	Female	Crizotinib	22 cycles	SD	PR brain metastasis
					Brigatinib	9 months	Rapid clinical response	
					Lorlatinib	1 year	PR	Complete surgical resection after 1 year Lorlatinib
Styczewska^36^	*TFG::ROS1*	Tongue	7	Female	Crizotinib	4 months	PR	Complete surgical resection after Treatment
Mai et al.^37^	*TFG::ROS1*	Right Lung	14	Male	Crizotinib	>8 months	PR	
Vassal g. et al.^38^	*Not reported partner::ROS1*	Na	Na	Na	Crizotinib	Na	PR	

As drug resistance may develop, regular CT scans to assess tumor dimensions and subsequent molecular re‐profiling in case of progression are crucial to detect resistance to the selective therapeutic pressure exerted by crizotinib. With brigatinib and lorlatinib, there are alternatives in case of progression. Unfortunately, all case reports cover only a short follow‐up period. Longitudinal follow‐up, including monitoring of *ROS1* fusion transcripts and detection of potential resistance mutations in *ROS1*, is necessary to evaluate and select the best targeted treatment for patients with IMTs. A modern MTB infrastructure for tracking individual patient trajectories including clinical and molecular follow‐up needs to be implemented, especially in the context of actionable targets.

## 5 CONCLUSION

Despite their typically protracted and less aggressive course, IMTs remain challenging both in diagnosis and treatment. NGS‐based identification of genomic alterations and predictive genetic biomarkers have become an integral part of diagnostic workup in precision oncology. This case once more highlights the importance of molecular diagnostics, the value of access to a multidisciplinary MTB with access to cutting‐edge technology and the resulting individualized treatment.

## AUTHOR CONTRIBUTIONS

**Sebastian Sommer:** Conceptualization (equal); data curation (lead); writing – original draft (lead); writing – review and editing (lead). **Maximilian Schmutz:** Conceptualization (equal); data curation (equal); writing – original draft (equal); writing – review and editing (equal). **Tina Schaller:** Data curation (supporting); investigation (supporting). **Michaela Kuhlen:** Investigation (supporting); resources (supporting). **Frank Jordan:** Data curation (supporting); resources (supporting). **Sebastian Dintner:** Data curation (supporting). **Patrick Mayr:** Data curation (supporting). **M. Monika Golas:** Resources (supporting). **Bruno Märkl:** Resources (equal). **Ralf Huss:** Resources (supporting). **Rainer Claus:** Conceptualization (equal); writing – review and editing (equal). **Bernhard Heinrich:** Conceptualization (lead); data curation (equal); writing – review and editing (equal).

## CONFLICT OF INTEREST STATEMENT

The authors hereby confirm that there are no conflicts of interest in connection with this article.

## ETHICS STATEMENT

The patient provided her written informed consent for publishing the case report. According to the regional law Bayerisches Krankenhausgesetz (BayKrG) in the version of 28 March 2007 (GVBl. S. 288, BayRS 2126‐8‐G Art. 27 Abs4), an ethical statement for retrospective analysis of the data is not necessary.

## Data Availability

The data on which the results and conclusions of this study are based are available on personal request from the corresponding author.
